# Impact of Severe Acute Respiratory Syndrome Coronavirus 2 (SARS-CoV-2) in the Nervous System: Implications of COVID-19 in Neurodegeneration

**DOI:** 10.3389/fneur.2020.583459

**Published:** 2020-11-16

**Authors:** Myosotys Rodriguez, Yemmy Soler, Marissa Perry, Jessica L. Reynolds, Nazira El-Hage

**Affiliations:** ^1^Department of Immunology and Nano-medicine, Herbert Wertheim College of Medicine, Florida International University, Miami, FL, United States; ^2^Department of Medicine, Jacobs School of Medicine and Biomedical Sciences, University at Buffalo, Buffalo, NY, United States

**Keywords:** SARS-CoV-2, COVID-19, neurological complications, inflammation, cytokines

## Abstract

Coronavirus Disease 2019 (COVID-19), caused by the Severe Acute Respiratory Syndrome coronavirus-2 (SARS-CoV-2), began in December 2019, in Wuhan, China and was promptly declared as a pandemic by the World Health Organization (WHO). As an acute respiratory disease, COVID-19 uses the angiotensin-converting enzyme 2 (ACE2) receptor, which is the same receptor used by its predecessor, SARS-CoV, to enter and spread through the respiratory tract. Common symptoms of COVID-19 include fever, cough, fatigue and in a small population of patients, SARS-CoV-2 can cause several neurological symptoms. Neurological malaise may include severe manifestations, such as acute cerebrovascular disease and meningitis/encephalitis. Although there is evidence showing that coronaviruses can invade the central nervous system (CNS), studies are needed to address the invasion of SARS-CoV-2 in the CNS and to decipher the underlying neurotropic mechanisms used by SARS-CoV-2. This review summarizes current reports on the neurological manifestations of COVID-19 and addresses potential routes used by SARS-CoV-2 to invade the CNS.

## Introduction

In December 2019, a pneumonia outbreak of unknown etiology was first reported in Wuhan, China. The etiology of the respiratory illness was attributed to a new strain of coronavirus and the disease was subsequently named Coronavirus Disease 2019 (COVID-19) by the World Health Organization (WHO) in February 2020. The novel strain responsible for COVID-19 was officially named as Severe acute respiratory syndrome coronavirus 2 (SARS-CoV-2). According to the WHO, as of July 10, 2020, globally there were ~12,322,395 cases of COVID-19 and 556,335 deaths confirmed (1). Coronaviruses are enveloped viruses that are comprised of a positive-sense single-strand RNA genome. Currently, there are seven strains of coronaviruses (CoV) that infect humans, including the 229E, NL63, OC43, HKU1, MERS-CoV, SARS-CoV, and SARS-CoV-2. SARS-CoV and 2, MERS-CoV, and HCoV-OC43 are classified as part of the betacoronavirus (βCoV) genus (2), and two of the genus, SARS-CoV and MERS-CoV, were responsible for the outbreaks in 2003 and 2012, respectively. In addition to the severe respiratory symptoms, SARS-CoVs can be associated with neurological symptoms in infected patients (3, 4). Despite evidence pointing to the possibility of central nervous system (CNS) invasion by coronaviruses, clinical data on the significance of CNS involvement in patients with COVID-19 are relatively scarce and most information are derived from SARS-CoV (5). However, emerging evidence suggests that COVID-19 infection could be implicated in damaging several organs, including the brain. During the early stages of the outbreak, reports indicated a high rate of olfactory and gustatory dysfunction in COVID-19 positive patients (6–9). Because these symptoms involve the cranial nerves, it is suspected that SARS-CoV-2 can trigger neurological manifestations. In addition, most coronaviruses share a similar viral structure and infection mechanism (10, 11). In the early 2000s, after the outbreak of SARS-CoV, virus was detected in postmortem brains recovered from SARS positive patients (12). SARS-CoV enters the host cells by binding to the angiotensin-converting enzyme 2 (ACE2) receptor, which is expressed in many different brain cells (13). Due to the genomic similarities between SARS-CoV and SARS-CoV-2 (14), and the shared affinity for the ACE2 receptor (15, 16), it is likely that SARS-CoV-2 can also target the brain, via receptor binding endocytosis/transcytosis. Thus, it is possible for COVID-19 positive patients to present with a severe infection that could ultimately manifest into neurological complications.

## Pathology of SARS-CoV-2 in The CNS

In patients infected with the SARS-CoV-2, neurological manifestations have been reported in several studies, ranging from minor common symptoms such as headache and anosmia to more severe and less common complications such as seizure and stroke (17). Since the ACE2 receptor used by SARS-CoV-2 can be expressed in vascular endothelium of the CNS, this can explain the neurotropic potential of the virus (18, 19). To date, only a few reports have confirmed detection of SARS-CoV-2 in the cerebrospinal fluid (CSF), while the key manifestations of SARS-CoV-2 remain predominantly in the lungs and in the immune system. Of note, is that the presence of SARS-CoV-2 is easily detected in the respiratory tract or the blood, allowing for a clear diagnosis of COVID-19. The severity and haste of the COVID-19 clinical course limit the accessibility for sampling and testing of CSF. However, several case studies have shown a clear link between severe COVID-19 infection and neurological manifestations, such as encephalitis, seizures, and cerebrovascular complications. Other common manifestations reported are dizziness, headaches, confusion, convulsions, and loss of consciousness. A retrospective study showed that 78 of 214 (36.4%) patients with laboratory-confirmed diagnosis of COVID-19 presented characteristic neurological manifestation of SARS-CoV-2 infection in hospitals from Wuhan, China (20). Elderly patients that are commonly presented with a more severe disease, are more likely to develop neurological symptoms compared to non-severe and younger patients. Similarly, a hospital in France reported that 49 of the 58 consecutive patients admitted for Acute respiratory distress syndrome (ARDS) due to COVID-19, presented neurological manifestations (21). The most characteristic neurological manifestations presented by these patients were encephalopathy, prominent agitation and confusion, and diffuse corticospinal tract signs. A case study that described the results of brain magnetic resonance imaging (MRI) from patients in the intensive care unit (ICU) with COVID-19 pneumonia, showed that 50 out of 235 ICU patients (21%) developed neurological symptoms (22). Brain MRIs were performed in 27 out of the 50 (54%) patients with neurologic symptoms, and 10 out of 27 (37%) patients presented cortical signal abnormalities. In some cases, the observed abnormal cortical signals were accompanied by cortical diffusion restriction, leptomeningeal enhancement, or cortical blooming artifact. CSF obtained in 5 out of 10 patients with cortical signal abnormalities, showed negative for SARS-CoV-2. The authors explained the difficulties in discerning between direct neurotropism of COVID-19 with others virus-related manifestations such as cytokine storm syndrome, hypoxia, subclinical seizures and encephalopathy (22). An early report in March 2020, described a 74-year-old male with past medical history of atrial fibrillation, cardioembolic stroke, Parkinson's disease, and chronic obstructive pulmonary disease (COPD) (23). The patient presented with severe alteration in mental status and was found to be encephalopathic. Cerebrospinal fluid studies did not show any evidence of CNS infection, however, due to the progression of his symptoms, the patient was tested for COVID-19 and was confirmed positive. Unfortunately, no further details were available in the case report. The authors concluded that SARS-CoV-2 does not cross the blood-brain barrier (BBB) and does not cause meningitis or encephalitis. This case report highlighted the importance of recognizing that encephalopathy may be a potential manifestation of COVID-19 (23). The first reported case of meningitis/encephalitis associated with SARS-CoV-2 was observed in a 24-year-old man, and was reported in April 2020 (24). The patient was brought to the hospital due to a convulsion accompanied by unconsciousness and was diagnosed with meningitis and viral pneumonia. Interestingly, the nasopharyngeal sample was negative for SARS-CoV-2 RNA, but was positive in the CSF sample. Moriguchi and colleagues declared the importance of this case because it shows that unconscious patients are potentially infected by SARS-CoV-2 and might cause the horizontal infection. The report warned the physicians of patients who have CNS symptoms that encephalitis may be the first indication, as well as respiratory symptoms, in identifying SARS-CoV-2 cases. Another case of meningitis, also reported in April 2020 by Duong et al., was detected in a 41-year-old female (25). Nasopharyngeal swab tested positive for SARS-CoV-2, but they were unable to send her CSF sample for PCR testing to confirm for COVID-19. While the authors reported of COVID-19 infection as an isolated case of meningoencephalitis without respiratory involvement, they couldn't directly confirm the presence of the COVID-19 virus in the CSF (25). In May 2020, an update on the case reported earlier by Duong et al. confirmed that the CSF sample was positive for SARS-CoV-2. This was the first case where SARS-CoV-2 infection was completely constrained to the CNS, with no association to other organs (26).

A potential association of COVID-19 with cerebrovascular diseases have been reported by several groups. A case of COVID-19–associated with acute necrotizing hemorrhagic encephalopathy was described in a female in her late fifties, although her CSF sample was never tested SARS-CoV-2 (27). In another case, two patients presented with COVID-19 and with neurological symptoms simultaneously, although their CSF samples repeatedly tested negative for the SARS-CoV-2 RNA (28). The first patient was reported with a case of aneurysmal subarachnoid hemorrhage of grade 3 in the Hunt and Hess scale, and the second patient was reported with ischemic stroke and massive hemorrhagic conversion. Goldberg et al. reported the first case of COVID-19-related to acute cerebrovascular disease in a 64-year-old man. The patient died after 3 days due to COVID-19-related complications, and no further clinical evaluations were performed (29). In another case report from a hospital in Belgium, 3 out of 31 patients with advance tumor-like brain lesions, tested positive with SARS-CoV-2 (30). All three patients suffered from diffuse intraparenchymal hemorrhage post-operatively, suggesting that an active infection may be related to a higher bleeding risk. In June 2020, a case series from King's College Hospital (KCH) in the United Kingdom, demonstrated five consecutive cases of predominantly lobar COVID-19-associated with intracerebral hemorrhage (ICH) (31). The cases were detected in patients with an average age of 52.2 years (range of 41–64 years): an age group that was particularly lower than expected for conventional ICH.

Seizures and epilepsy are less commonly reported neurological manifestations seen in COVID-19 patients. In a retrospective multicenter study from several hospitals in China, it was reported that 84 of 304 (27%) patients had brain insults or metabolic imbalances during the acute phase of COVID-19 infection, which is known to increase the risk of seizures (32). However, no new onset of seizures or status epilepticus were seen. The authors suggested that although the risk of suffering from seizures during acute COVID-19 illness is minimum, severely ill patients with underlying conditions have a higher risk of seizure occurrence. In another case, a 54-year-old woman who tested positive for COVID-19, was admitted with a case of disturbance of consciousness and seizures. MRI from the patient's brain and spine showed a new onset of multiple, non-enhancing demyelinating lesions (33). Similarly, the CSF sample tested negative for SARS-CoV-2. According to the authors, a possible cause for the observed neurological damage is due to the severe pneumonia that leads to CNS hypoxia triggering neurological damage. Taken together, these reports provide a potential association of SARS-CoV-2 with neurological manifestations in patients with COVID-19. With the pandemic limiting hospital resources and the haste of the disease, it is possible that other similar cases of neurological symptoms in patients with COVID-19 were not confirmed or reported because of the inability to detect the virus in CSF samples, or because of rapid clinical deterioration in patients.

In order to investigate the coexistence of coronaviruses within the CNS, a study by Niu and colleagues, created a fatal encephalitis virus attached to a luciferase reporter, that can be infected in animal model. Viral titer in mice infected with the HCoVOC43-ns2DelRluc, was reported by bioluminescence imaging (34). The authors showed viral infection in the CNS (brain and spinal cord), validating that this mouse model can be used as a tool to study coronaviruses in the CNS (34). The advantage of using this mouse model is that viral titer is measured in real time, allowing for the study of virus progression through the mouse's body. Although the viral-infected mouse model was used for a known neurotropic coronavirus, the model can be a useful tool to study SARS-CoV-2, as the virus has been observed in the frontal lobe suggesting that it can migrate to the CNS (18). Given the difficulties of obtaining samples and performing extensive analysis in hospitals, these reports and emerging experimental studies are critical for understanding the potential neurotropism and neurovirulence of SARS-CoV-2.

## Association of COVID-19 With Pre-Existing Chronic Neurological and Immune-Mediated Diseases

Alarming reports describing the elderly population as more susceptible to poor outcomes with COVID-19 (35–37), this has sparked growing concerns about COVID-19 and chronic diseases comorbidities. The tendency of severe COVID-19 illness in older individuals has been strongly associated with age-related loss of protein homeostasis (38). Elderly individuals often experience a decrease in the activation of the autophagy pathway and other stress responses, that can result in an accumulation of protein aggregates: a characteristic of neurodegenerative disorders associated with aging, for example Parkinson's disease (PD). Thus, suggesting that a possible long-term consequence of SARS-CoV-2 infection can be the accelerated aging in tissues affected by the virus (38). In addition, patients with advanced PD may be at higher risk of suffering from pneumonia due to possible underlying respiratory dysfunction (39). Thus, it is likely that suffering from PD may increase the propensity of severe respiratory complications and poor outcomes during COVID-19 infection. Helmich and Bloem also addressed how higher levels of psychological stress and adaptive difficulties during the COVID-19 pandemic may worsen several PD symptoms (39). Another neurodegenerative disease characterized by neuroinflammation is Alzheimer's Disease (AD). The excessive inflammatory and immune responses caused by SARS-CoV-2 infection may hasten the progression of inflammatory neurodegeneration in the brain among the elderly, increasing the risk for AD and the susceptibility to severe outcomes after SARS-CoV-2 infection (37).

Although studies on SARS-CoV-2 and chronic neurological diseases are limited, previous studies on other coronavirus implicate systemic infection with other neuronal syndromes, such as multiple sclerosis (MS) and acute disseminated encephalomyelitis (40, 41). MS patients have an increased risk of infections compared to others, therefore these patients could be at higher risk of COVID-19 (42). However, several case reports suggest that MS patients develop mild disease and can recover from COVID-19 (43–45). This is likely due to the immunosuppressant or immunomodulatory therapy used for MS on modulating the cytokine storm caused by SARS-CoV-2. A study showed that patients taking the immunosuppressant, fingolimod, can mount an effective immune response against SARS-CoV-2, via antibody-secreting cells, despite having low levels of T-lymphocyte subsets (46). In terms of acute disseminated encephalomyelitis, a case report from 2004, showed the detection of human coronavirus in the CSF of a child presumed to have acute disseminated encephalomyelitis. Authors suggested that coronavirus may represent an important etiological agent in the pathogenesis of demyelinating disease (41). Other reports have associated Guillain-Barré syndrome (GBS) as a consequence of the peripheral nervous system injury due to the hyperinflammation induced by SARS-CoV-2. Zhao described a case report of COVID-19 associated with acute GBS (47), in which they speculated that SARS-CoV-2 could have been the causative agent for the development of GBS. Moreover, the first appearance of GBS symptoms in the patient coincided with the period of SARS-CoV-2 infection. Thus, it is possible that GBS associated with SARS-CoV-2 infection might follow a parainfectious profile. Another case report described the possible correlation between acute COVID-19 infection and GBS in a 71-year-old male patient (48). The patient was presented with a severe form of acute polyradiculoneuritis with prominent demyelinating features and a subsequent diagnosis of GBS associated with COVID-19 was made. Taken together, these studies suggest that SARS-CoV-2-mediated inflammation may accelerate the progression of pre-existing neurological conditions. It remains unknown if patients suffering from immune-mediated inflammatory diseases are at higher risk of COVID-19 severity. However, special attention should be given to a possible cross-reactive of immunity between SARS-CoV-2 antigens and host tissues that could develop or worsen the immune system disorders.

## Potential Routes of SARS-CoV-2 Invasion To the CNS

The exact mechanism used by SARS-CoV-2 to invade the CNS has not been fully elucidated. Given the similarity between SARS-CoV and SARS-CoV-2, the infection mechanism reported in previous outbreaks may also be pertinent for SARS-CoV-2. It is well-documented that the host receptor ACE2, used by SARS-CoV-2 for viral entry, is expressed in numerous human cells, such as respiratory and intestinal epithelial cells, endothelial cells, kidney cells, alveolar monocytes/macrophages, and cerebral neurons and glia (49–52). In fact, a study provided further evidence that the SARS-CoV-2 spike (S) protein binds to ACE2 receptor with higher affinity when compared to the S-protein expressed by SARS-CoV (53).

There are two major routes proposed for SARS-CoV-2 invasion into the brain: the hematogenous and neuronal retrograde routes (2, 50, 54, 55). In the hematogenous route, the virus invades the brain by infecting the epithelial cells of the blood-cerebrospinal fluid barrier and/or the endothelial cells of the blood-brain-barrier (BBB). This route of invasion was supported by Paniz-Mondolfi et al. (18), who detected the presence of SARS-CoV-2 in neural and capillary endothelial cells in postmortem frontal lobe tissue from a COVID-19 patient. In the neuronal retrograde route, the virus invades the CNS by infecting peripheral neurons. Many COVID-19 positive patients, especially with mild cases, suffer from hypogeusia (reduced ability of taste), as well as anosmia (decreased sense of smell) (7, 56–58). Hypogeusia observed after SARS-COV-2 infection could result from direct injury of the glossopharyngeal nerve and the vagus nerve by the virus (59). In addition, SARS-CoV-2 can directly infect the olfactory sensory neurons in the olfactory epithelium and then be transported into the CNS through the olfactory nerve. Reports using animal models of spontaneously hypertensive rats (SHRs) and normotensive Wistar-Kyoto (WKY) control rats, have shown ACE2 expression in the nucleus of the solitary tract (60, 61), which points to the central cause of dysgeusia and a possible neuro-invasive route by continuous local or retrograde vagal axonal transport. Another report on the HCoV-OC43 showed the presence of structural viral material along axons in both *ex vivo* animal model of human coronaviruses (HCoV) neuropathogenesis and *in vitro* neuronal cell culture (62). Suggesting the use of the retrograde neuronal transport by HCoV-OC43 to facilitate locally the infection of neuronal cells from the olfactory nerve to the brain stem.

In addition to a direct infection of SARS-CoV-2 in the CNS, it is likely that numerous cases of neurological manifestations are indirect and most likely due to immune activation or hypoxia injury. SARS-CoV-2 infection induces excessive and sustained cytokine and chemokine responses, known as the cytokine storm. The proinflammatory state induced by the cytokine storm is characterized mainly by the dysregulated production of cytokines, such as interleukin (IL)-1, IL-6, and tumor necrosis factor (TNF)-α, that can trigger the development of virus induced systemic inflammatory response syndrome (SIRS) or SIRS-like immune disorders (63). This phase of hyperinflammation may cause disruption of the BBB, activation of glial cells, demyelination and severe encephalopathy (64). Studies have demonstrated that high level expression of inflammatory cytokines, such as IL-6, of IL-1β, interferon (IFN)-γ, interferon gamma-induced protein 10 (IP-10), TNF-α, and the chemokine, monocyte chemoattractant protein 1 (MCP-1) can correlate with the severity of COVID-19 symptoms (65–68). Several reports suggest that the high mortality of COVID-19 might be due to the hyperinflammation driven by SARS-CoV-2 (66, 69, 70). Hyperinflammation can also induce apoptosis of endothelial and epithelial cells, damaging the pulmonary microvascular and alveolar epithelial cell barriers and causing vascular leakage and alveolar edema, eventually leading to hypoxia in the body (68). Severe COVID-19 positive patients suffer from respiratory distress that leads to systemic hypoxemia. Hypoxic conditions caused by lung injury contributes to high neuro-susceptibility to COVID-19 (59). Therefore, it can be expected that patients with COVID-19 may develop seizures as a consequence of hypoxia, metabolic derangements, or organ failure (71). Evidence suggests that SARS-CoV-2 can induce CNS hypoxia that leads to an increase in anaerobic metabolism required to trigger neurological damages, such as demyelinating lesions (33). Hypoxic neurotoxicity and subsequent damages to the CNS can be observed without the direct presence of SARS-CoV-2 in the brain. Another study reported the occurrence of delirium in COVID-19 positive patients as a potentially early symptom of hypoxia associated with severe respiratory failure, or of an infectious spread to the CNS mediated by neuro-invasive mechanisms of the coronavirus (72). In conclusion, despite solid evidences of the neuro-invasive characteristics of human coronaviruses, the mechanism of neuro-invasion of SARS-CoV-2 remains to be established.

## Potential Mechanisms of SARS-CoV-2 Pathology in The Brain

Genomic similarities shared by SARS-CoV-2 and other coronavirus, especially the human SARS-CoV have facilitated investigations of this novel virus. A bioinformatics analysis reported that the overall genome of SARS-CoV-2 shares 82% nucleotide identity with SARS-CoV (73). The viral Spike protein, responsible for cell entry, contains two subunits, including the S1 and S2 subunit. Accordingly, the S2 subunit of SARS-CoV-2 shares 99% amino acid homology with the S subunit derived from SARS-CoV. Whereas, the S1 subunit shares around 70% homology with SARS-CoV. The S1 subunit contains the N-terminal domain (NTD) and a receptor-binding domain (RBD), responsible for the direct binding of the virion to the host cellular ACE2 receptor (74). Once inside the CNS, SARS-CoV-2 can infect and trigger the activation of several brain cells. The most abundant cells in the brain are the astrocytes. They provide homeostasis and play an important role in the secretion of inflammatory molecules and neurotrophic factors (75–77). ACE2 receptors are expressed in brainstem and cerebellum astrocytes from rat (78), and may be a target for SARS-CoV-2 infection in the brain. A recent study analyzed two biomarkers for CNS injury, glial fibrillary acidic protein (GFAP) and neurofilament light chain (NfL) in the plasma of 47 patients with mild, moderate or severe COVID-19 (79). NfL is an axonal structural protein expressed in neurons that is released as a result of axonal injury and axonal neurodegeneration (80, 81), while GFAP is expressed in astrocytes and it is markedly upregulated in CNS injury and inflammation (82–84). Patients with severe COVID-19 had significantly higher concentrations of GFAP and NfL in plasma than controls, and GFAP was also increased in patients with moderate COVID-19. Interestingly, in the severe group, expression levels of plasma GFAP decreased from the initial to the last follow-up sampling, whereas NfL expression increased from initial to the last sampling. This study suggests that astrocytosis (astrocytes activation) could be a characteristic of moderate and severe stages of COVID-19, while neuronal injury may reflect the severe stages of the disease (79).

Microglia are considered the prototypic tissue-resident macrophage-like innate immune cells of the CNS (85). They are involved in chemotaxis, phagocytosis, antigen presentation, and cytokine production, however, impaired or infected microglia contributes to the secretion of neuroinflammation and the process of neurodegeneration (86). Several studies have linked microglia activation and dysfunction with coronaviruses infection. A study investigated the role of microglia in responding to viral infection using mice infected with the neuro-attenuated rJ2.2 strain of the murine coronavirus, mouse hepatitis virus (MHV). Microglia were required at the earliest stages of infection to control viral replication and to increase host survival (87). While depletion of microglia resulted in a more rapid viral replication, increased infection of neurons, ineffective T cell responses, and evasion of the immune response by the virus. This study demonstrated that microglia have a critical role in the immune response to viral infection and protection from viral encephalitis. Another study determined that encephalitic murine coronavirus (MHV-A59) can be transmitted to the brain by intranasal or intracerebral contact, resulting in the activation of the innate immune system and the secretion of pro-inflammatory cytokine by microglia and type I astrocytes (but not types II and III) (88). Suggesting that microglia and type I astrocytes form part of the CNS glymphatic system and mediate the innate immune reaction, specifically by the release of cytokines following an encephalitic coronavirus infection. It can be expected that the virus elicits distinctive tropism for each cell type in the brain. A report showed that two coronaviruses, the neurotropic virus (MHV-A59) and the non-neurotropic virus (MHV-2) infect astrocytes and microglia at the same titer in mice (89). However, cytokine mRNA profiles were different between astrocytes and microglia cultures. Infection with MHV-A59 increased the secretion of proinflammatory immune responses in astrocytes and microglia when compared to infection with MHV-2. These findings suggest that the neurovirulence of coronavirus is associated with the virus capacity to induce proinflammatory immune response by glia in the CNS.

Prolonged cytokine signaling, as observed in COVID-19 pathology can induce cellular senescence. Senescent cells, characterized by sustained cell cycle arrest and the production of a distinct senescence-associated secretory phenotype (SASP), such as cytokines and chemokines (90, 91), are commonly detected in neurodegenerative diseases. A study suggested the use of lithium, as a senolytic agent, to selectively induce apoptosis of senescent cells, as a potentially therapy to treat COVID-19 positive patients (92). They reported that low doses of lithium decreased the release of IL-6 and IL-8 and suppressed amyloid-β (Aβ) increased SA β-gal staining in induced pluripotent stem cells (iPSCs)-derived astrocytes, all hallmarks of cellular senescence. IL-6 increase during the cytokine storm is often correlated to COVID-19 severity, suggesting that cellular senescence and the production of SASP as a potential mechanism of SARS-CoV-2 injury to the brain.

As mentioned above, the autophagy pathway is necessary to remove aggregated or damaged cytoplasmic proteins and organelles. This pathway is constitutively active in the brain, and disruption of the pathway can affect the intercellular communication of axons, causing dendrites damage, loss of synapses, and consequently contributing to neurodegeneration (93–95). Autophagy is also involved in the removal of pathogens (Xenophagy) (96–98) and a key player in the replication of different genus of viruses and in the secretion of inflammatory molecules from glial cells (99–105). The replication of coronaviruses depends on the formation of replication complexes double-membrane vesicles derived from the endoplasmic reticulum (ER) (106, 107). Because the autophagy pathway also relies on ER-derived membranes for autophagosomes formation (108, 109), possible usage of the autophagy machinery by coronavirus to support viral replication has been suggested. A report showed the co-localization of SARS-CoV viral proteins with the endogenous protein marker of autophagosomes, LC3. Authors also suggested that SARS-CoV may use cellular autophagy to facilitate the formation of the replication complexes on double-membrane vesicles (110). On the other hand, a report showed no significant differences in SARS-CoV infection in mouse embryonic fibroblasts overexpressing the human ACE2 receptor from autophagy-deficient ATG5 knockout mice when compared to wild-type mice (111). To date, there are no reports available that link SARS-CoV-2 to the autophagy pathway in brain cells. A non-peer-reviewed preliminary report showed that SARS-CoV-2 reduced the autophagic flux and hampered autophagosome/lysosome fusion efficiency in bronchial epithelial cells NCI-H1299 and monkey kidney cells (VeroFM) *in vitro*, while the induction of autophagy reduced SARS-CoV-2 propagation *in vitro* (112). Although the exact role of autophagy in SARS-CoV-2 cell entry and infection in the brain remains undetermined, the autophagy pathway regulates an effective inflammatory response and prevents prolonged hyperinflammation (113). Thus, suggesting that autophagy could play a role in SARS-CoV-2 infection or in SARS-CoV-2-induced inflammatory response. More studies are needed to investigate the potential role of autophagy in SARS-CoV-2 pathology, specifically in cells from the CNS.

## Conclusions and Future Perspectives

Published reports validate that infection by SARS-CoV-2 is not solely confined to the lungs. Current evidence concurs that SARS-CoV-2 can impact the brain by direct and indirect injury. As investigations related to COVID-19 are still in progress, more studies should be conducted using pre-clinical animal models and clinical samples from SARS-CoV-2 positive patients and autopsies of the COVID-19 victims. Furthermore, it is imperative to improve the timely evaluation of neurological symptoms and early analysis of the CSF in COVID-19 positive patients. Increased awareness of neurological complications related to SARS-CoV-2 infection is crucial to improve the prognosis of severely ill patients. It is necessary to keep accurate registries and to follow-up with those affected and who have recovered from COVID-19. Monitoring the long-term consequences of SARS-CoV-2 infection in the CNS, including long-term psychological and neurocognitive implications is also necessary.

## Author Contributions

**Figure 1:**
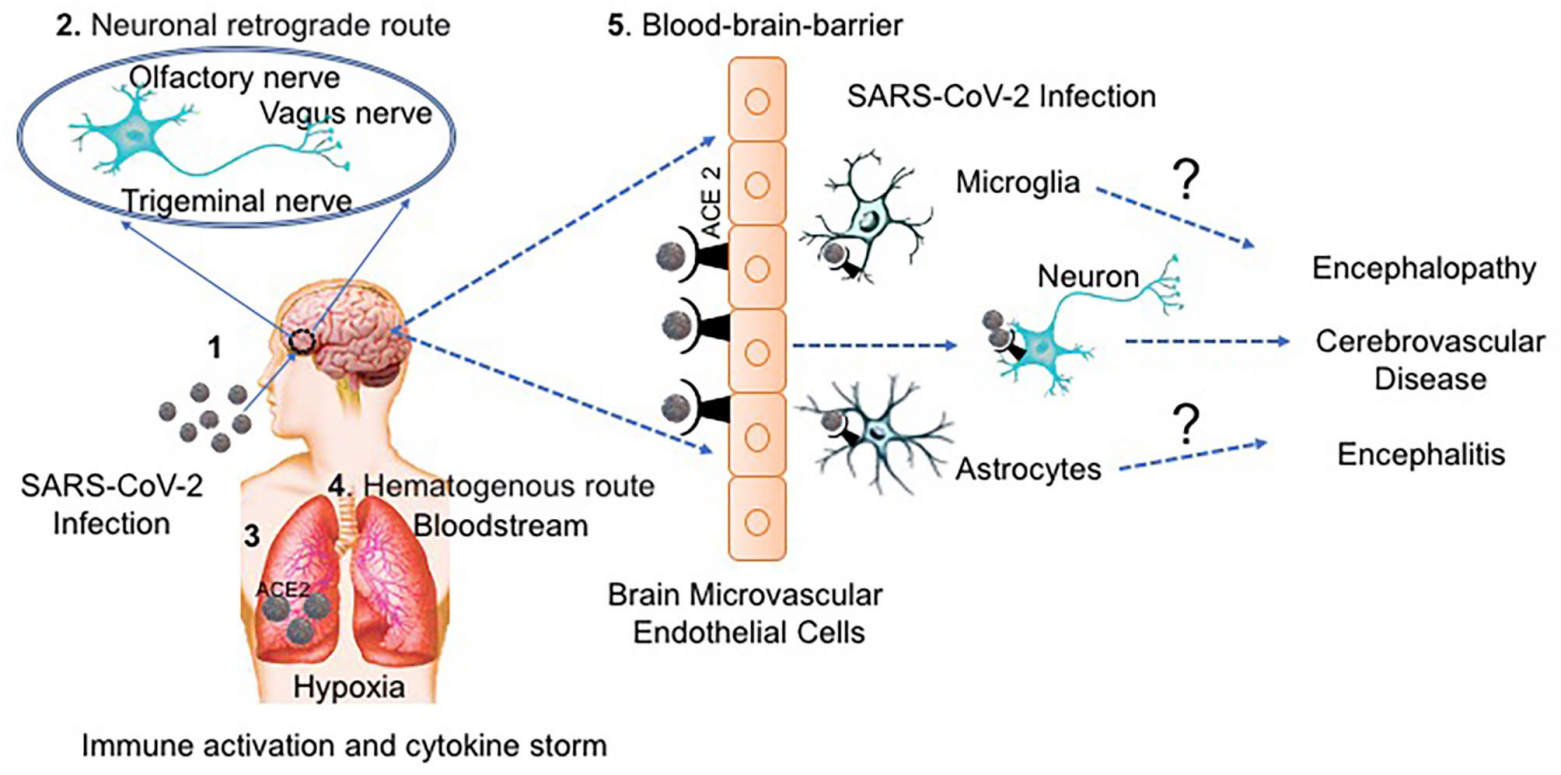
The potential routes of SARS-CoV-2 neurological invasion. (1) SARS-CoV-2-containing droplets are deposited on the mucosa membranes through intranasal delivery, (2) Accordingly, the virus can infect the peripheral nervous system using the neuronal retrograde route until it reaches the brain. (3) SARS-CoV-2 binds to ACE2 receptors expressed on epithelial cells and enters the respiratory tract, (4) Alternatively, SARS-CoV-2 can enter the bloodstream by the hematogenous route and (5) transmigrate the BBB through receptor (ACE2) binding transcytosis/endocytosis on the endothelial monolayers. Once SARS-CoV-2 reaches the brain, it can infect the brain cells, probably the astrocytes and microglia or can infect the neurons directly. SARS-CoV-2 can infect and activate both astrocytes and microglia causing a release virion, viral proteins, host-inflammatory molecules and other neurotoxins that cause neuronal damage and disease. Furthermore, infection with SARS-CoV-2 can indirectly trigger neurological injury through an exaggerated immune response and by the hypoxic environment caused by respiratory distress.

MR: manuscript outline, preparation of the draft manuscript, and editing of the manuscript. YS, MP, and JR: assisted in writing the manuscript. NE-H: preparation of Figure 1, writing, critical reading, and editing of the manuscript. All authors: contributed to the article and approved the submitted version.

## Conflict of Interest

The authors declare that the research was conducted in the absence of any commercial or financial relationships that could be construed as a potential conflict of interest.
